# Targeted alpha-therapy using [Bi-213]anti-CD20 as novel treatment option for radio- and chemoresistant non-Hodgkin lymphoma cells

**DOI:** 10.18632/oncotarget.817

**Published:** 2013-02-24

**Authors:** Mareike Roscher, Inis Hormann, Oliver Leib, Sebastian Marx, Josue Moreno, Erich Miltner, Claudia Friesen

**Affiliations:** ^1^ Centre for Biomedical Research, University of Ulm, Helmholtzstrasse 8/1, Ulm, Germany; ^2^ Institute of Legal Medicine, University of Ulm, Prittwitzstrasse 6, Ulm, Germany; ^3^ Isotope Technologies Garching GmbH (ITG), Lichtenbergstrasse 1, Garching, Germany

**Keywords:** non-Hodgkin lymphoma, targeted alpha-therapy, anti-CD20, Bismuth-213, radioresistance, apoptosis

## Abstract

Radioimmunotherapy (RIT) is an emerging treatment option for non-Hodgkin lymphoma (NHL) producing higher overall response and complete remission rates compared with unlabelled antibodies. However, the majority of patients treated with conventional or myeloablative doses of radiolabelled antibodies relapse. The development of RIT with alpha-emitters is attractive for a variety of cancers because of the high linear energy transfer (LET) and short path length of alpha-radiation in human tissue, allowing higher tumour cell kill and lower toxicity to healthy tissues. In this study, we investigated the molecular effects of the alpha-emitter Bi-213 labelled to anti-CD20 antibodies ([Bi-213]anti-CD20) on cell cycle and cell death in sensitive and radio-/chemoresistant NHL cells. [Bi-213]anti-CD20 induced apoptosis, activated caspase-3, caspase-2 and caspase-9 and cleaved PARP specifically in CD20-expressing sensitive as well as in chemoresistant, beta-radiation resistant and gamma-radiation resistant NHL cells. CD20 negative cells were not affected by [Bi-213]anti-CD20 and unspecific antibodies labelled with Bi-213 could not kill NHL cells. Breaking radio-/chemoresistance in NHL cells using [Bi-213]anti-CD20 depends on caspase activation as demonstrated by complete inhibition of [Bi-213]anti-CD20-induced apoptosis with zVAD.fmk, a specific inhibitor of caspases activation. This suggests that deficient activation of caspases was reversed in radioresistant NHL cells using [Bi-213]anti-CD20. Activation of mitochondria, resulting in caspase-9 activation was restored and downregulation of Bcl-x_L_ and XIAP, death-inhibiting proteins, was found after [Bi-213]anti-CD20 treatment in radio-/chemosensitive and radio-/chemoresistant NHL cells. [Bi-213]anti-CD20 seems to be a promising radioimmunoconjugate to improve therapeutic success by breaking radio- and chemoresistance selectively in CD20-expressing NHL cells via re-activating apoptotic pathways through reversing deficient activation of caspases and the mitochondrial pathway and downregulation of XIAP and Bcl-x_L_.

## INTRODUCTION

A quite novel and promising treatment option for B-cell non-Hodgkin lymphoma (NHL) are radioimmunotherapies (RIT) using monoclonal antibodies (mabs) as vehicle to selectively target malignant cells with the coupled radionuclide [1].

CD20, a non-glycosylated 33 kDa transmembrane phosphoprotein appearing to be involved in the regulation of B-cell growth and differentiation [2], is due to its characteristics a promising target for immunotherapy of B-cell malignancies using chimerical (mouse/human) anti-CD20-antibodies (Rituximab/anti-CD20) [2]: It has a low membrane turnover and furthermore, it is expressed stably on more than 90% of all malignant NHL cells, normal B-cells but not on stem cells, mature plasma cells or other tissues [3, 4]. Rituximab is the first monoclonal antibody licensed for immunotherapy of NHL and is applicated as monotherapy or in combination with chemotherapeutics [5, 6]. Despite the promise of therapy with unmodified antibodies and the improvement in overall response rates and survival of patients with NHL, only 6% to 20% of patients achieve complete remissions while significant numbers of relapsed patients can be observed [5, 6]. RIT could be proven promising to overcome the limitations of unconjugated antibodies: The anti-CD20-radioimmunoconjugates [I-131]tositumomab (Bexxar^®^) and [Y-90]ibritumomab-tiuxetan (Zevalin^®^) produce higher overall response and complete remission rates compared with unlabelled antibodies [7]. The majority of patients treated with conventional or myeloablative doses of radiolabelled anti-CD20-antibodies, however, also relapse [7]. The development of RIT with alpha-emitters such as Bi-213 is promising for treatment of a variety of cancers and is attractive because of the high linear energy transfer (LET) and short path length of alpha-radiation in human tissue, allowing higher tumour cell kill and lower toxicity to healthy tissues [8-12].

Apoptosis can be stimulated via the external pathway induced by the specific binding of death ligands to their receptors (e.g. CD95/CD95 ligand) or by the mitochondrial pathway [13]. Activation of either pathway leads to the activation of the caspase cascade in which initiator caspases (like caspase-2 and caspase-9) as well as effector caspases (e.g. caspase-3 or caspase-7) are involved leading to the concerted destruction of the cell [14].

Resistances against chemotherapeutics and/or radiation in cancer therapy, which are one the primary causes for therapeutic failure, can be a result of changes in the apoptotic pathways [15-17]. The Bcl-2 family of proteins including pro-apoptotic members like Bax and anti-apoptotic members like Bcl-x_L_ regulate the integrity of the outer mitochondrial membrane. [18-21]. The majority of human cancers harbour high levels of inhibitor of apoptosis proteins (IAPs), such as the well characterised X-linked IAP (XIAP) [22]. Chemo-/Radioresistance of NHL are often associated with the overexpression of different molecules like XIAP or Bcl-x_L_ [22]. Targeting these proteins by inhibition or downregulation is a novel approach in cancer therapy [23]. Recently, it was shown that the alpha-emitter Bi-213 labelled to an anti-CD45-antibody has the capacity to abrogate chemo- and radioresistance in leukaemia cells via caspase activation and activation of the mitochondrial apoptotic pathway *in vitro* [24].

In general, the increasing employment of so-called targeted alpha-therapies (TAT) leads to the question how these particles exhibit their cytotoxicity in cancer cells and which signalling cascades are involved – but only few studies have been published [24-29]. Therefore, we investigated the molecular effects of the alpha-emitter Bi-213 labelled to anti-CD20 antibodies ([Bi-213]anti-CD20) on the cell cycle and cell death in radio-/chemosensitive as well as in radio-/chemoresistant NHL cells. We clarified the molecular mechanisms for cell death induction and overcoming of radio-/chemoresistance. Our study demonstrates that after a G2-phase arrest, [Bi-213]anti-CD20 leads to apoptosis induction via activation of caspases using the mitochondrial pathway in sensitive as well as in radio- and chemoresistance in NHL B-cells. In addition, [Bi-213]anti-CD20 induces apoptosis in NHL which are resistant to anti-CD20 antibodies or to antibodies labelled with Y-90. [Bi-213] bound to anti-CD20 seems to be a promising therapeutic strategy in the treatment of NHL especially if conventional therapeutic modalities failed.

## RESULTS

### [Bi-213]anti-CD20 induces cell death specifically in CD20-positive NHL cells

Anticancer drugs, beta- as well as gamma-radiation are known to induce apoptosis and to activate apoptotic pathways in leukaemia, lymphoma and solid tumours [13, 16, 24]. Furthermore, also the radioimmunoconjugate [Bi-213]anti-CD45 induces cell death via apoptosis in CD45-positive leukaemia cells [24].

**Figure 1:**
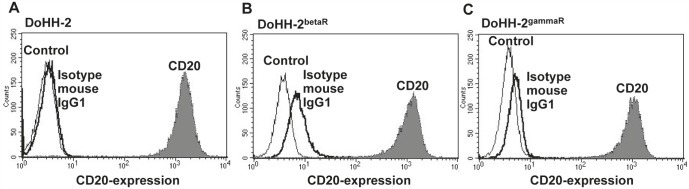
NHL cells express CD20 on their cell surface (A,B,C) DoHH-2 cells (A), DoHH-2 cells resistant to beta-irradiation (DoHH-2^betaR^) (B) or resistant to gamma-irradiation (DoHH-2^gammaR^) (C) were stained with mouse anti-CD20-PE-IgG1 antibodies and analyzed by flow cytometry. Untreated cells (Control) are exhibited as thin solid curves, the isotype matched controls detecting unspecific binding of the antibodies as thick solid curves (Isotype mouse IgG1) and the mouse anti-CD20-PE-IgG1 antibodies stained cells as grey filled curves (CD20).

As monoclonal anti-CD20-antibodies alone or as radioimmunoconjugate labelled with Y-90 or I-131 are employed in the treatment of NHL with quite good results [7], we wanted to determine the cytotoxic potential of anti-CD20-antibodies in *in vitro* settings applied as TAT approach using the alpha emitter Bi-213. The NHL cell line DoHH-2 (Figure 1A) as well as the beta-radiation resistant cell line DoHH-2 (DoHH-2^betaR^) (Figure 1B) and gamma-radiation resistant cell line DoHH-2 (DoHH-2^gammaR^) (Figure 1C) express comparable amounts of the CD20-antigen on their surface as shown by flow cytometry analysis. Therefore, these cell lines can be directly targeted using the anti-CD20-radioimmunoconjugate.

**Figure 2:**
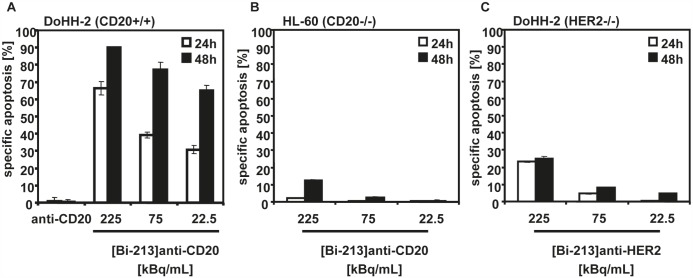
[Bi-213]anti-CD20 kills CD20-positive NHL cells specifically (A) [Bi-213]anti-CD20 induces apoptotic cell death in CD20-positive (CD20+/+) NHL cells. The NHL cell line DoHH-2 was incubated with the indicated activity concentrations of [Bi-213]anti-CD20 or unlabelled anti-CD20 (anti-CD20), respectively. The amount of unlabelled anti-CD20 antibodies corresponds to the one used for the highest activity concentration of the radioimmunoconjugate [Bi-213]anti-CD20. (B) [Bi-213]anti-CD20 leads to minor cell kill in CD20-negative (CD20−/−) HL-60 cells. The AML cell line HL-60 was treated with different activity concentrations of [Bi-213]anti-CD20 as indicated. (C) anti-HER2 does not kill HER2-negative (HER2−/−) NHL cells strongly. HER2-negative DoHH-2 cells were incubated with [Bi-213]anti-HER2 with activity concentrations as indicated. (A,B,C) 24h (white columns) and 48h (black columns) after application of the respective radioimmunoconjugate, the percentages of apoptotic cells were measured by FSC/SSC-analysis. The percentage of specific cell death was calculated as follows: 100 - [experimental dead cells (% - spontaneous dead cells in medium (%)] / [100 % - spontaneous dead cells in medium (%)]. Columns, mean of triplicates; bars, SD <10%.

First, we analyzed whether [Bi-213]anti-CD20 induces cell death in the NHL B-cell line DoHH-2 and which type of cell death can be induced by targeted alpha-radiation. Therefore, we treated the DoHH-2 cells with different activity concentrations (225, 75, 22.5kBq/mL) of [Bi-213]anti-CD20 using a specific activity of ~4MBq/μg antibody. 24h and 48h after applying the radioimmunoconjugates, a time and dose-dependent induction of apoptosis could be detected in DoHH-2 cells (Figure 2A). The unlabelled anti-CD20-antibody which was used in a concentration of about 56ng/mL equivalent to the amount of radiolabelled antibody applicated for 225 kBq/mL [Bi-213]anti-CD20 showed no cytotoxicity (Figure 2A). Next, we assessed whether the radioimmunconjugate induced cell death is specifically triggered by [Bi-213]anti-CD20 or whether it is an unspecific side-effect of the applied Bi-213. Therefore, we treated the CD20-negative AML cell line HL-60 with [Bi-213]anti-CD20 using comparable activity concentrations and specific activities (Figure 2B). In the non-targeted HL-60 cells (Figure 2B) only a slight induction of cell death could be observed in comparison to the CD20-expressing DoHH-2 cells (Figure 2A), demonstrating that [Bi-213]anti-CD20 induces apoptosis specifically in CD20-expressing cells. In addition, we analyzed the cytotoxic potential of [Bi-213]anti-HER2-antibody ([Bi-213]anti-HER2) on HER2-negative DoHH-2 cells which hence cannot target treated DoHH-2 cells specifically (Figure 2C). 24h and 48h after treating DoHH-2 cells using 225, 75 and 22.5kBq/mL [Bi-213]anti-HER2 with a specific activity of ~4MBq/μg antibody, respectively, just a small fraction of cells could be observed to be apoptotic (Figure 2C). This demonstrates that [Bi-213]anti-CD20 specifically targets CD20-positive NHL leading only to minor damage in surrounding healthy tissue.

### [Bi-213]anti-CD20 breaks radio- and chemoresistance in CD20-positive NHL cells

**Figure 3:**
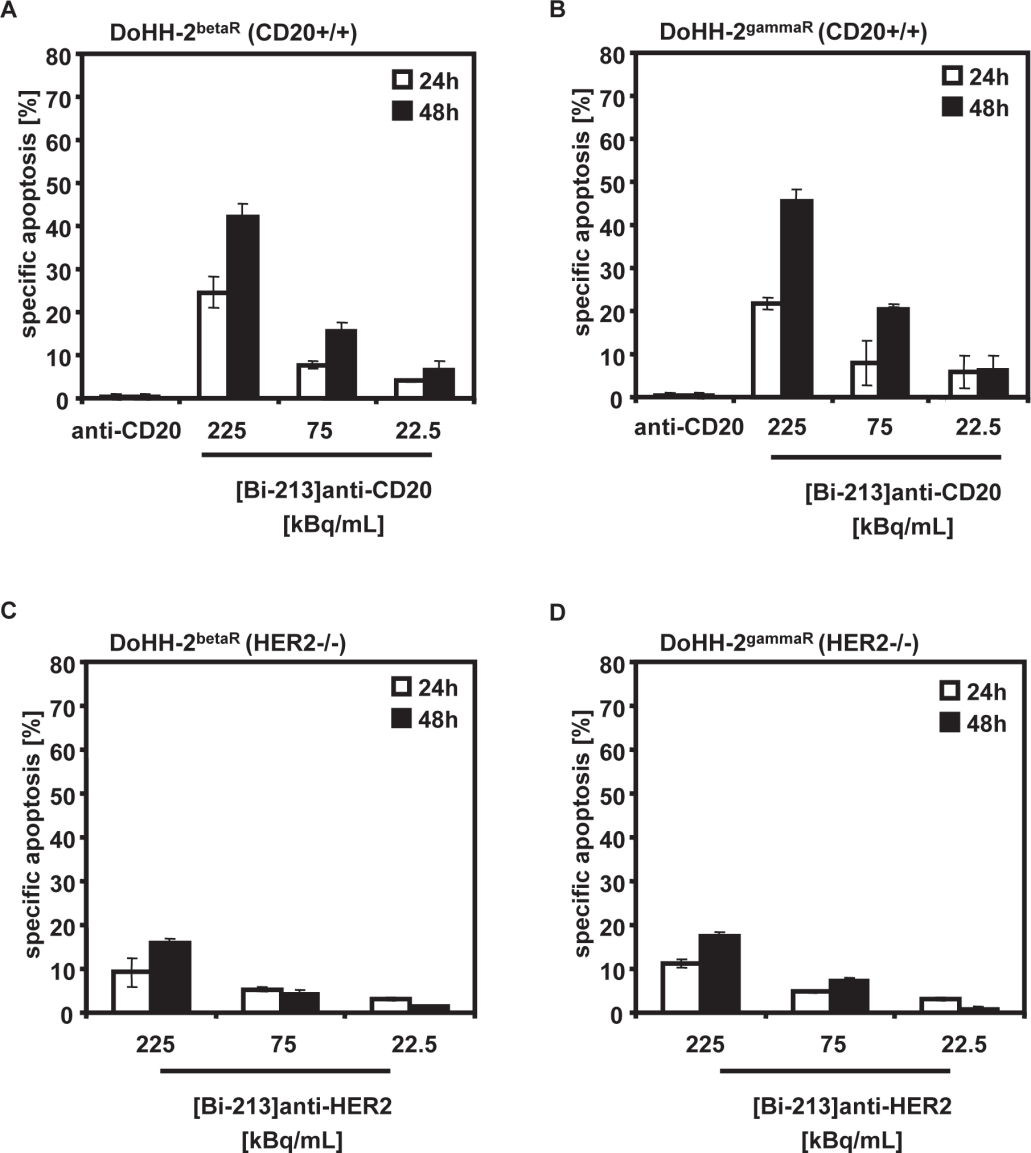
[Bi-213]anti-CD20 breaks radio- and chemoresistance in CD20-positive NHL cells (A,B) [Bi-213]anti-CD20 overcomes radio- and chemoresistance in CD20-positive (CD20+/+) beta-radiation resistant (DoHH-2^betaR^) (A) and gamma-radiation resistant DoHH-2 cells (DoHH-2^gammaR^) (B). DoHH-2 cells resistant against up to 5Gy Y-90 (DoHH-2^betaR^) (A) or resistant against up to 5Gy Cs-137 (DoHH-2^gammaR^) (B) and cross-resistant to different chemotherapeutics were incubated with different activity concentrations of [Bi-213]anti-CD20 as indicated or unlabelled anti-CD20 (anti-CD20), respectively. The amount of unlabelled anti-CD20 antibodies corresponds to the one used for the highest activity concentration of the radioimmunoconjugate [Bi-213]anti-CD20. (C,D) [Bi-213]anti-HER2 does not induce a strong cell death in HER2-negative (HER2−/−) DoHH-2^betaR^ (C) or HER2-negative (HER2−/−) DoHH-2^gammaR^ cells (D), respectively. DoHH-2^betaR^ (C) or DoHH-2^gammaR^ (D) were incubated with activity concentrations of [Bi-213]anti-HER2 as indicated. (A,B,C,D) After 24h (white columns) and 48h (black columns), the percentage of apoptotic cell death was measured by FSC/SSC-analysis. The specific cell death was calculated as described in Figure 2. Columns, mean of triplicates; bars, SD <10%.

[Bi-213]anti-CD45 could be shown to overcome chemo- and radioresistances in CD45-expressing leukaemia cells [24]. We analyzed whether also [Bi-213]anti-CD20 has the potential to abrogate resistances and to re-activate defective apoptotic signalling pathways in radio- and chemoresistant CD20-positive NHL cells. Therefore, we treated the beta-radiation resistant DoHH-2 (DoHH-2^betaR^) (Figure 3A) and gamma-radiation resistant DoHH-2 (DoHH-2^gammaR^) (Figure 3B) cells which are cross-resistant to different chemotherapeutics and resistant to anti-CD20 labelled with the beta-emitter Y-90 ([Y-90]anti-CD20), with 225, 75, 22.5kBq/mL of [Bi-213]anti-CD20 using a specific activity of ~4MBq/μg antibody. A strong induction of apoptosis was detected in DoHH-2^betaR^ and DoHH-2^gammaR^ cells after 24h and 48h (Figure 3A,B). Applicating the corresponding amount of unlabelled anti-CD20 antibodies showed no cytotoxicity. This indicates that [Bi-213]anti-CD20 overcomes gamma-resistance and beta-radiation resistance such as Y-90, [Y-90]anti-CD20 resistance, as well as chemoresistance. The unspecific radioimmunoconjugate [Bi-213]anti-HER2 led to smaller extents of cell kill in the HER2-negative DoHH-2^betaR^ and DoHH-2^gammaR^ cells (Figure 3C,D). This suggests that [Bi-213]anti-CD20 breaks beta- and gamma-radiation resistance as well as chemoresistance specifically in targeted CD20-positive NHL cells.

### [Bi-213]anti-CD20 leads to changes in the cell cycle distribution in CD20-positive NHL cells

**Figure 4:**
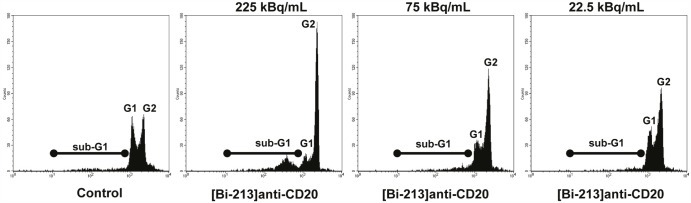
[Bi-213]anti-CD20 arrests the cell cycle in G2/M-phase in CD20-positive NHL cells CD20-positive (CD20+/+) DoHH-2 cells were left untreated (Control) or incubated with different activity concentrations of [Bi-213]anti-CD20 as indicated. 20h after applying the radioimmunoconjugate [Bi-213]anti-CD20, the DNA content and cell cycle distribution could be analysed using flow cytometry. The apoptotic cells were determined by analysis of hypodiploid nuclei (black line, sub-G1). Cells in G1-phase have nuclei with a diploid DNA content (G1, nuclei in G1-phase) and cells in G2-phase nuclei with a tetraploid DNA content (G2, nuclei in G2/M-phase).

Ionizing radiation interrupts the normal cell cycle progression in mammalian cells by activating cell cycle checkpoints. Once these are activated, cells are arrested either in G1/S-, S- or G2/M-phases of the cell cycle until the damage is repaired. If no repair is possible apoptosis is induced [30]. We measured propidium iodide stained nuclei via flow cytometry in order to measure the subdiploid DNA content of cells (sub-G1) - a central hallmark of apoptotic cells - and cell cycle distribution of DoHH-2 cells (Figure 4). We found a G2/M-phase arrest and an increase in the fraction of cells with subdiploid DNA content depending on activity concentrations (Figure 4).

### [Bi-213]anti-CD20 reversed deficient caspase activation in CD20-positive radio-/chemoresistant NHL cells

**Figure 5:**
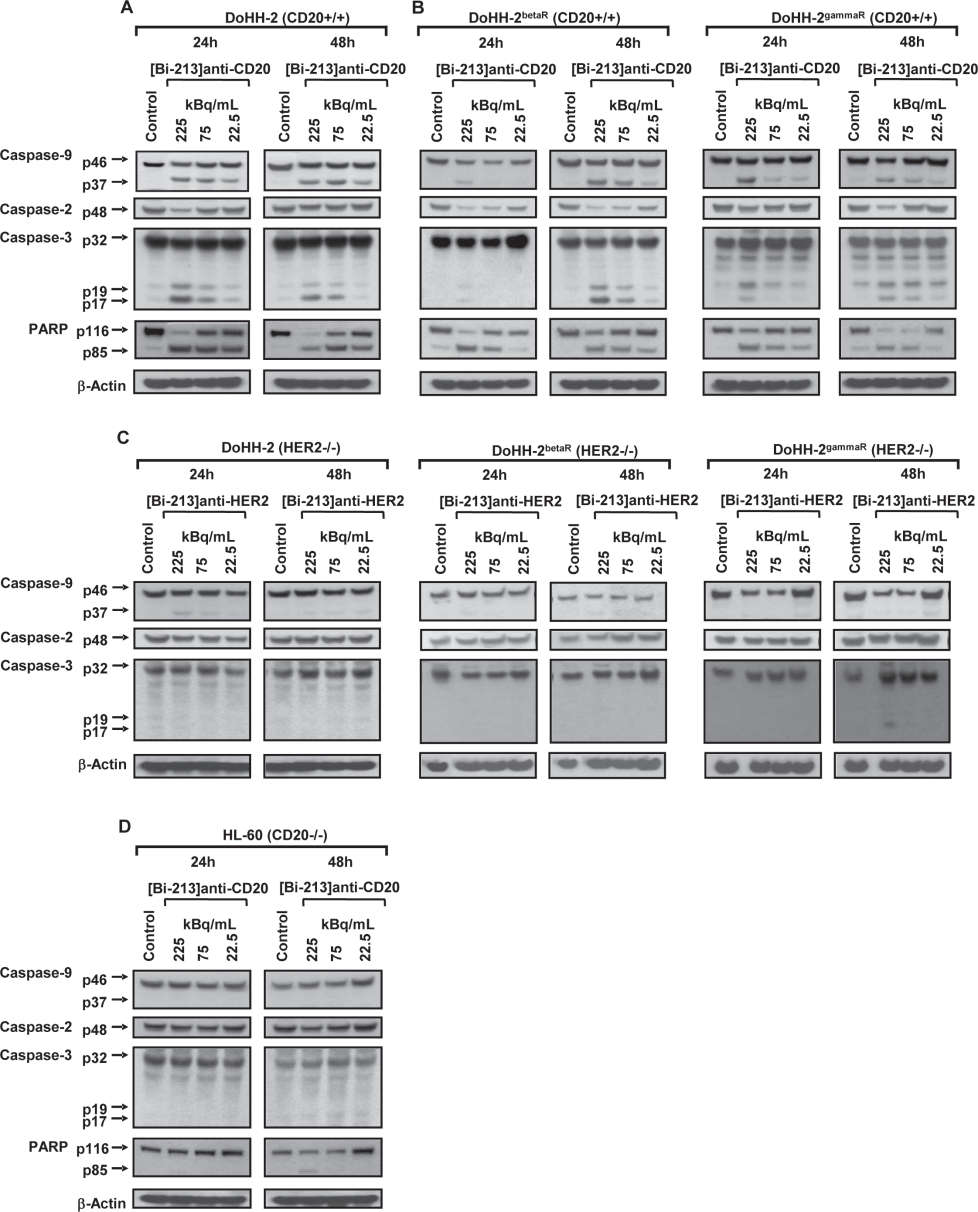
[Bi-213]anti-CD20 restores deficient caspase activation and apoptotic pathways in CD20-positive NHL cells specifically (A,B) [Bi-213]anti-CD20 leads specifically to the activation of the caspase cascade. The CD20-positive (CD20+/+) NHL cell line DoHH-2 (A) as well as the CD20-positive (CD20+/+) NHL beta-radiation resistant cell line DoHH-2^betaR^ (B) or gamma-radiation resistant cell line DoHH-2^gammaR^ (B) were either left untreated (Control) or were incubated with different activity concentrations of [Bi-213]anti-CD20 as indicated. (C) The HER2-negative (HER2−/−) NHL cell line DoHH-2 (C) as well as the HER2-negative (HER2−/−) NHL beta-radiation resistant cell line DoHH-2^betaR^ (C) or gamma-radiation resistant cell line DoHH-2^gammaR^ (C) were either left untreated (Control) or were incubated with different activity concentrations of [Bi-213]anti-HER2 as indicated. (D) The CD20-negative (CD20−/−) cell line HL-60 was either left untreated (Control) or was incubated with different activity concentrations of [Bi-213]anti-CD20 as indicated. (A,B,C,D) 24h and 48h after applying the antibodies labelled with Bi-213, protein lysates were isolated and Western-blot analyses for caspase-9, -2, -3 and PARP performed. Downregulation of procaspase-2 was detectable at ~48kDa. The active fragment of caspase-9 was detected at ~37kDa, the active fragment of caspase-3 at ~19kDa and ~17kDa and PARP cleavage at ~85kDa. Equal protein loading was controlled using an anti-β-Actin-antibody.

To clarify which molecular mechanisms are involved in [Bi-213]anti-CD20 induced apoptosis and which are restored in breaking radio-/chemoresistance, we examined the induced signalling pathways by Western-blot analyses. After treating the radio-/chemosensitive DoHH-2 cells (Figure 5A) as well as the radio-/chemoresistant DoHH-2^betaR^ and DoHH-2^gammaR^ cells (Figure 5B) for 24h and 48h with activity concentrations of 225, 75 and 22.5kBq/mL [Bi-213]anti-CD20, the effector caspase-3 (Figure 5A,B) and the initiator caspase-9 (Figure 5A,B) could be activated in a dose- and time-dependent manner. Caspase-2 was activated, resulting in a minor reduction of the zymogen. Furthermore, PARP, the prototype caspase substrate [31], could be shown to be effectively processed (Figure 5A,B). This suggests that [Bi-213]anti-CD20 activates caspases in radio-/chemosensitive as well as in radio-/chemoresistant DoHH-2 NHL cells, indicating that defective activation of caspases was reversed in the radio-/chemoresistant DoHH-2 cells. In contrast to [Bi-213]anti-CD20 targeting in radio-/chemosensitive DoHH-2 and radio-/chemoresistant DoHH-2^gammaR^ or DoHH-2^betaR^ cells, the unspecific radioimmunoconjugate [Bi-213]anti-HER2 could not activate caspase-3, caspase-9, and caspase-2 (Figure 5C). Only at the highest activity concentration (225kBq/mL) a slight activation of caspases could be observed (Figure 5C). Similar data were obtained analysing the effects of [Bi-213]anti-CD20 on the AML cell line HL-60 which does not express the CD20-antigen (Figure 5D). These results demonstrate that [Bi-213]anti-CD20 activates caspases and apoptotic pathways selectively in targeted CD20 positive NHL cells.

**Figure 6:**
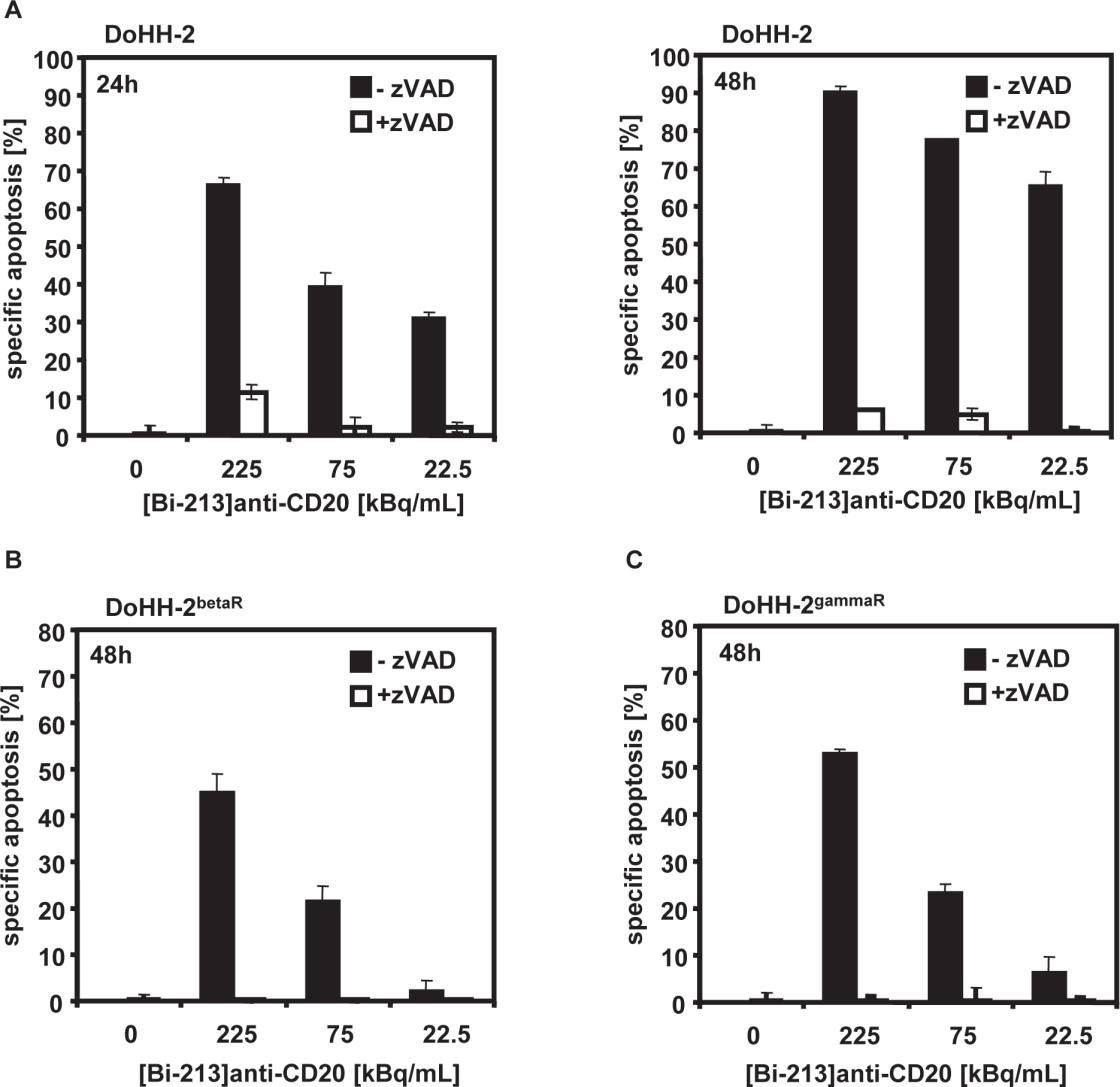
[Bi-213]anti-CD20 induced apoptosis depends on caspase activation (A,B,C) The CD20-positive (CD20+/+) radiosensitive DoHH-2 (A), the CD20-positive (CD20+/+) beta-radiation resistant DoHH-2^betaR^ (B), and the CD20-positive (CD20+/+) gamma-radiation resistant DoHH-2^gammaR^ cells (C) pre-treated with 50μM of the broad-range caspase inhibitor zVAD.fmk (+zVAD; white columns) or without pre-treatment (-zVAD; black columns) were incubated with different activity concentrations of [Bi-213]anti-CD20 and time points as indicated. Apoptosis induction was measured at different time points using flow cytometry. The percentage of specific cell death was calculated as described in Figure 2. Columns, mean of triplicates; bars, SD <10%.

The importance of caspase activation in cell death induction of [Bi-213]anti-CD20 was further analyzed with the pan-caspase inhibitor zVAD.fmk. When co-treated with zVAD.fmk and [Bi-213]anti-CD20, the fraction of dead cells in the radio-/chemosensitive DoHH-2 (Figure 6A) as well as in the radio-/chemoresistant DoHH-2^betaR^ (Figure 6B)and DoHH-2^gammaR^ (Figure 6C) cell lines diminished nearly completely. This underlines that apoptosis induction depends on the activation of caspases in radio-/chemosensitive and in radio-/chemoresistant NHL cells. The deficient activation of caspases was reversed in resistant NHL cells after [Bi-213]anti-CD20 treatment.

**Figure 7:**
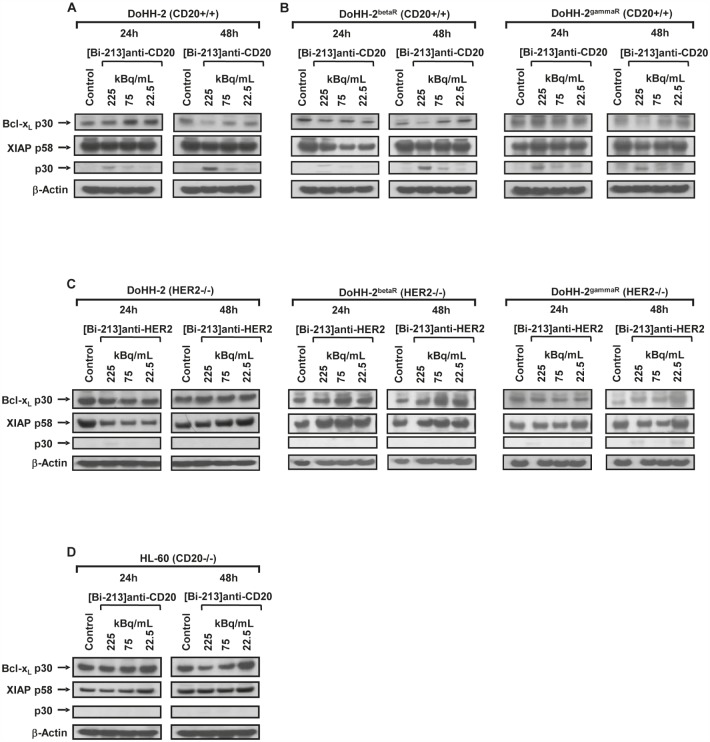
[Bi-213]anti-CD20 induces apoptosis and breaks radio-/chemoresistance by downregulation of XIAP and Bcl-x_L_ in NHL cells (A,B) The CD20-positive (CD20+/+) NHL cell line DoHH-2 (A) as well as the CD20-positive (CD20+/+) beta-radiation resistant DoHH-2^betaR^ (B) and the CD20-positive (CD20+/+) gamma-radiation resistant DoHH-2^gammaR^ (B) were either left untreated (Control) or incubated with different activity concentrations of [Bi-213]anti-CD20 as indicated. (C) The HER2-negative (HER2−/−) NHL cell line DoHH-2 (C) as well as the HER2-negative (HER2−/−) beta-radiation resistant cell line DoHH-2^betaR^ (C) or the HER2-negative (HER2−/−) gamma-radiation resistant cell line DoHH-2^gammaR^ (C) were either left untreated (Control) or were incubated with different activity concentrations of [Bi-213]anti-HER2 as indicated. (D) The CD20-negative (CD20−/−) cell line HL-60 was either left untreated (Control) or was incubated with different activity concentrations of [Bi-213]anti-CD20 as indicated. (A,B,C,D) Western-blot analyses were performed for Bcl-x_L_ and XIAP after 24h and 48h. Downregulation of Bcl-x_L_ was detected at ~30kDa and XIAP was detected at ~58kDa and its degradation product at ~30kDa. Equal protein loading was controlled using an anti-β-Actin-antibody.

IAPs and Bcl-x_L_ play a critical role in resistance of tumours to conventional therapies [22, 32, 33]. The anti-apoptotic Bcl-x_L_ prevents permeabilisation of the mitochondrial membrane during apoptosis induction and Bcl-x_L_ overexpression is linked to apoptosis resistance of NHL cells. Among the IAP family proteins, XIAP displays the strongest anti-apoptotic properties and inhibits apoptosis signalling by binding to active caspase-3 and by preventing caspase-9 activation [34]. Inhibition of XIAP by XIAP-inhibitors or Bcl-x_L_ by using Bcl-x_L_-antisense oligonucleotides sensitizes cancer cells for chemotherapeutic drugs or radiation [32]. After incubation with different activity concentrations (225, 75, 22.5 kBq/mL) of [Bi-213]anti-CD20, we detected a downregulation of Bcl-x_L_, downregulation of XIAP and cleavage of XIAP in radio-/chemosensitive DoHH-2 (Figure 7A) cells as well as in the radio-/chemoresistant DoHH-2^betaR^ and DoHH-2^gammaR^ cells (Figure 7B). In addition, treating the HER2-negative DoHH-2 NHL cells with [Bi-213]anti-HER2 (Figure 7C) or the radio-/chemoresistant DoHH-2^betaR^ and DoHH-2^gammaR^ cells with [Bi-213]anti-HER2 (Figure 7C) or the CD20-negative HL-60 cells with [Bi-213]anti-CD20 (Figure 7D) did not lead to significant changes in the protein levels of Bcl-x_L_ or XIAP. These results indicate that [Bi-213]anti-CD20 reversed deficient activation of caspases and apoptotic pathways via downregulation of XIAP and Bcl-x_L_ specifically in CD20-positive NHL cells.

## DISCUSSION

B-cell NHL comprise a heterogeneous group of lymphoproliferative malignancies with different biological behaviour and treatment response [35]. In many NHL patients, the natural course of disease is one of increasing resistance ultimately leading to treatment failure [36]. Immunotherapy using monoclonal antibodies like Rituximab targeting the B-cell-antigen CD20 has been widely accepted for the treatment of NHL [37]. Despite encouraging clinical results with anti-CD20-antibodies, however, the majority of patients relapse [6, 7]. Recently, RIT with [I-131]tositumomab and [Y-90]ibritumomab-tiuxetan has emerged as promising treatment option for NHL as it could be observed that the radioimmunoconjugates lead to higher overall response and complete remission rates if compared with unlabelled antibodies [7, 38-40]. Nevertheless, only 1.5% and 17% of the total energy for Y-90 and I-131 are estimated to be deposited in a tumour of 200μm in diameter, while the remainder is deposited in surrounding healthy tissues leading to dose-limiting toxicities [41]. Alpha-emitters like Bi-213 and At-211 with high linear energy transfer (LET) and short path length in human tissue, allow higher specific tumour cell kill and lower toxicity to healthy tissues [42, 43].

[Bi-213]anti-CD45 could be shown to induce apoptosis in CD45-expressing leukaemia cells whereby its efficiency to kill leukaemia cells is much greater than that of comparable activities of beta- and gamma-radiation [24] which might lead to a reduction of the overall applicated concentration of radioactivity. Furthermore, it is a well-established concept that the amount of the vehicle can be reduced drastically if employed in radiotherapies in comparison to monotherapies. We could demonstrate that the radioimmunoconjugate [Bi-213]anti-CD20 induced apoptosis specifically in CD20-positive NHL DoHH-2 cells whereas the unlabelled antibody could not exhibit any cytotoxicity due to its low concentration. [Bi-213]anti-HER2, which could not target DoHH-2 cells, displayed only minor cytotoxic effects even if high activities were applied. In addition, [Bi-213]anti-CD20 could not exhibit its destructive potential in CD20-negative HL-60 cells. This suggests that [Bi-213]anti-CD20 kills selectively CD20-positive NHL cells with limited side-effects. These data are in line with previously published studies. Vandenbulcke [28] could show that [Bi-213]anti-CD20 induced significantly higher cell death rates in cells to which the antibody had a higher binding capacity. In a second study [27], they demonstrated that [Bi-213]anti-CD20 killed chronic lymphatic leukaemia cells more effectively than external gamma-irradiation underlining the importance of this novel therapeutic approach [27]. Similar results were also found in CD45-expressing leukaemia cells treated with [Bi-213]anti-CD45 [24]. Survival analyses of B-cell lymphoma cell cultures and haematopoietic progenitor cells, derived from the bone marrow, indicated a high tumour cell-to-normal bone marrow cell toxicity ratio of [At-211]anti-CD20 [44, 45]. Although the bone marrow cells are inherently sensitive to alpha-radiation, a much higher cell kill in the tumour cells could be shown underlining once more the specific targeting of the radioimmunoconjugate [44]. As irradiation of healthy cells and tissues cannot be avoided in RIT, the applied activities have to be chosen to induce only acceptable damages to these. Nevertheless, pre-targeted TAT using anti-CD20-antibody-streptavidin constructs and Bi-213-radiolabelled biotin in a mouse NHL xenograft model was well-tolerated with no treatment-related mortalities and a high therapeutic efficacy resulting in a significant prolongation of survival in all treated mice [41]. Due to the pre-targeting, the tumour-to-background ratio could be increased minimizing non-specific radiation exposure to healthy organs [41].

Major problems in the treatment of NHL are relapses and resistances which might develop during anti-cancer treatment. Alpha-particles have been shown to overcome chemo- and radioresistance in leukaemia cells [11, 24]. We could demonstrate that [Bi-213]anti-CD20 can also induce cell death in beta-radiation- and gamma-radiation-resistant CD20-positive NHL cells cross-resistant to a variety of anti-cancer drugs indicating that [Bi-213]anti-CD20 breaks radioresistance and chemoresistance in targeted NHL cells. The highest absorbed dose of radioactivity in the studies corresponds to 1.5 Gy whereas the cells are resistant against 5 Gy of beta- or gamma-radiation, respectively, underlining once more the possibility of reducing the total applicated dose of radioactivity. Our findings suggest that TAT is a promising approach for the treatment of relapsed and radio-/chemoresistant NHL.

Radiation and chemotherapeutic drugs induce apoptosis by activating apoptotic pathways in cancer cells [13]. Deficient induction of apoptotic pathways was found in cancer cells resistant to conventional therapies [17]. Our study demonstrates that [Bi-213]anti-CD20 triggers the intrinsic pathway of apoptosis leading ultimately in the recruitment of the caspase cascade in targeted CD20-positive NHL cells. In addition, [Bi-213]anti-CD20 reverses deficient activation of caspases in targeted radio-/chemoresistant CD20-positive NHL cells through mitochondria activation. After treatment with [Bi-213]anti-CD20, caspase-3 activation and hence PARP cleavage were observed in radio-/chemosensitive as well as in radio-/chemoresistant CD20-positive NHL cells. Formation of the cytochrome-c/Apaf-1/caspase-9-containing apoptosome complex plays a critical role in mitochondrial activation [46]. We found that caspase-9 was activated after treatment with [Bi-213]anti-CD20 in radio-/chemosensitive as well as in radio-/chemoresistant CD20-positive NHL cells, demonstrating that the mitochondrial pathway is involved in apoptosis induction and deficient activation of caspase-9 and of mitochondria is reversed in radio-/chemoresistant CD20-positive NHL cells after [Bi-213]anti-CD20 treatment.

In many studies, the clinical relevance of the expression of apoptosis regulatory proteins was analyzed using immunohistochemistry or gene expression profiling [47]. These data show that NHL cells display a defective apoptosis regulation provoked by the overexpression of the anti-apoptotic proteins Bcl-x_L_ and XIAP [48, 49]. XIAP appears to be one of the strongest inhibitors of the apoptotic machinery and suppresses apoptosis by preventing activation of caspase-3 and caspase-9 [50]. The anti-apoptotic Bcl-x_L_ prevents permeabilisation of the mitochondrial membrane during apoptosis induction. Treatment with [Bi-213]anti-CD20 mediated the downregulation of XIAP as well as Bcl-x_L_, underlining that [Bi-213]anti-CD20 induced cell death depends on XIAP and Bcl-x_L_ in NHL cells and restores deficient activation of apoptotic pathways. This implies that [Bi-213]anti-CD20 therapies might improve the therapeutic success of NHL patients with resistances against conventional treatment modalities due to altered expression levels of XIAP and Bcl-x_L_.

Taken together, our study reveals that [Bi-213]anti-CD20 induces apoptosis selectively in CD20-expressing NHL cells using the mitochondrial pathway. [Bi-213]anti-CD20 is a promising radioimmunoconjugate to improve therapeutic success by breaking radio- and chemoresistance in CD20-expressing NHL cells via re-activating apoptotic pathways through reversing deficient activation of caspases and downregulation of XIAP and Bcl-x_L_.

## METHODS

### Cell culture

The CD20-positive human *NHL* B-cell line DoHH-2 and the CD20-negative acute myeloid leukaemia (AML) cell line HL-60 were obtained from DMSZ (Braunschweig, Germany). The cells were grown in suspension in RPMI supplemented with 1 mmol/L glutamine (Invitrogen, Karlsruhe, Germany), 1% penicillin/streptomycin (Invitrogen), 25 mmol/L HEPES (Biochrom AG, Berlin, Germany) and 10% FCS (Lonza, Verviers, Belgium) at 37°C, 5% CO_2_. Radiation resistant cell lines were generated tolerating 5 Gy of gamma-irradiation (Cs-137; DoHH-2^gammaR^) or 5 Gy of beta-irradiation (Y-90; DoHH-2^betaR^), respectively. Both radioresistant cell lines are cross-resistant to a subset of chemotherapeutics such as doxorubicin, cisplatin, and etoposide.

Prior to incubation with the radiolabelled antibodies, the cells were seeded in a density of 10^5^ cells/mL.

### Antibodies

The recombinant humanized anti-CD20-mab Rituximab (MabThera^®^, Roche, Mannheim, Germany) consists of a human IgG1 kappa constant region, with a variable region isolated from a murine anti-CD20-antibody specific for the CD20-antigen overexpressed on NHL cells [3, 4]. *In vivo*, it exhibits a low immunogenic potential [51]. The antibody was chelated with CHX-A``-DTPA and stored at 4°C.

The recombinant humanized mab Trastuzumab (Herceptin^®^, Roche) was chelated with CHX-A``-DTPA and stored at 4°C. This antibody (anti-HER2) is immunoreactive against the epidermal growth factor receptor HER2 and thereby inhibits the proliferation and survival of expressing cells [52].

### Radionuclides and radiolabelling of antibodies

Bi-213 with a half-life of 45.6 min decays by a branched pathway by alpha-/beta-emissions to stable Bi-209 [42]. Of the emitted energy 80% is deposited by alpha-particles with energies of 8.4 MeV (79%) and 5.9 MeV (1%) [42]. Bi-213 for the antibody radiolabelling was derived from an Ac-225/Bi-213 generator system. For the purification of the radioimmunoconjugates PD10 desalting columns (GE Healthcare, Munich, Germany) were used. Quality controls were performed via ITLC. Activities were applied to the cells using 225, 75, and 22.5 kBq/mL of Bi-213 labelled to the respective antibodies with specific activities of ~4 MBq/μg.

### Determination of apoptosis and cell cycle analysis

24h or 48h after applying the radioimmunoconjugates, quantification of apoptosis and cell cycle analysis were done by flow cytometry as described [24, 53, 54]. To determine apoptosis, cells were lysed with Nicoletti buffer containing 0.1% sodium citrate plus 0.1% Triton X-100 and 50 μg/mL propidium iodide at 4°C [53]. The percentage of apoptotic cells was measured by hypodiploid DNA (subG1) content [53] or forward scatter/side scatter analysis [54] using flow cytometry (FACSCalibur, Becton Dickinson, Heidelberg, Germany).

### Determination of CD20-expression on the cell surface

The NHL cell lines DoHH-2, DoHH-2^betaR^ and DoHH-2^gammaR^ were stained with 10μl phycoerythrin (PE)-labelled mouse anti-CD20-IgG1 antibody according to the manufacturer's instructions (Biozol, Eching, Germany). Immunofluorescence analyses were performed using flow cytometry. Mouse IgG1-antibodies (Southern Biotech, Birmingham, USA) as isotype control were used to assess the unspecific binding of the anti-CD20-PE-antibody.

### Caspase inhibition by zVAD.fmk

To determine the role of caspase activation in cell death induction, the cells were pre-treated for one hour with 50μM of the pancaspase inhibitor zVAD.fmk (z-Val-D,L-Asp-fluoromethylketone, Bachem, Heidelberg, Germany). After 24h or 48h an analysis of the cell viability was performed by FSC/SSC measurements.

### Western-blot analysis

Prior to the performance of immunoassays whole cell lysates were generated as previously described [24]. The proteins of interest were probed using rabbit-anti-PARP (1:1000, Roche), rabbit-anti-caspase-3, rabbit-anti-caspase-9 (both 1:1000, Cell Signaling, Boston, USA), rabbit-anti Bcl-x_L_ (1:500, Santa Cruz, Heidelberg, Germany), as well as mouse-anti-caspase-2, mouse-anti-XIAP (both 1:1000, BD Transduction Laboratories). For detection enhanced chemiluminescence was employed after incubating the membranes with horseradish peroxidase labelled goat-anti-rabbit or goat-anti-mouse IgG (1:5000, Santa Cruz), respectively. Mouse-anti-β-Actin (1:5000, Sigma, Seelze, Germany) was used as loading control.
